# An uncommon side effect of a very commonly used medication: A case report on hydrocortisone‐induced bradycardia

**DOI:** 10.1002/ccr3.4678

**Published:** 2021-08-16

**Authors:** Alvin Oliver Payus, Ann Chee Chai, Norlaila Mustafa

**Affiliations:** ^1^ Medicine Based Department Faculty of Medicine and Health Science Universiti Malaysia Sabah Kota Kinabalu Malaysia; ^2^ Department of Medicine Universiti Kebangsaan Malaysia Medical Centre (UKMMC) Cheras Malaysia

**Keywords:** bradycardia, corticosteroid, hydrocortisone

## Abstract

Sinus bradycardia is a rare but important side effect of high‐dose hydrocortisone. It is a self‐limiting condition that recovered spontaneously upon stopping the medication and did not recur with other types of corticosteroids.

## Graphical abstract

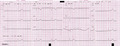

## 1 INTRODUCTION

Sinus bradycardia is a rare adverse effect of corticosteroid. Here, we report a case of a young woman who developed severe sinus bradycardia after given 200 mg of intravenous hydrocortisone and reverted back to normal sinus rhythm after stopping the medication and did not recur with another type of corticosteroid.

Corticosteroid is a potent immune‐suppressing and anti‐inflammatory medication, making it a very effective treatment modality for autoimmune and other inflammatory conditions such as acute exacerbation of bronchial asthma, acute gouty arthritis, multiple sclerosis, and nephrotic syndromes. Nevertheless, the use of corticosteroids is associated with several adverse effects where some are more common than others. Among the commonly reported adverse effects are hypertension, hyperglycemia, osteopenia, electrolyte imbalance such as hypokalemia, and increased susceptibility to infections. Cardiac arrhythmias such as sinus bradycardia are uncommonly seen adverse effects of corticosteroid, which was first reported in 1986.^1^ This uncommon side effect of a very commonly used medication is the center of discussion in this case report.

## 2 CASE REPORT

**FIGURE 1:**
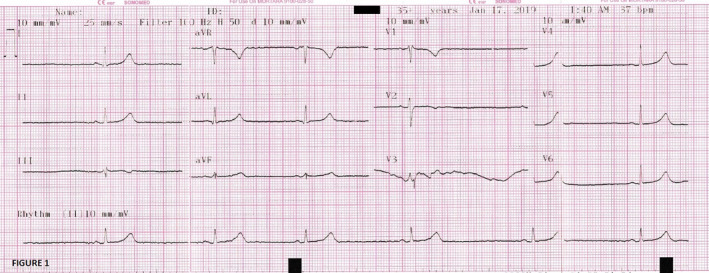
Electrocardiogram done after given intravenous hydrocortisone 200 mg showed sinus bradycardia with the heart rate of 30 beats per minute, partial right bundle branch blocked, and no evidence of coronary ischemia

A 40‐year‐old lady with an underlying systemic lupus erythematosus and lupus nephritis since 2002 currently under control with oral prednisolone 10 mg once daily, oral azathioprine 50 mg once daily, and oral hydroxychloroquine 250 mg twice daily presented to the emergency department with a low‐grade fever and multiple episodes of vomiting and watery diarrhea for the past 3 days. There was no joint pain, no alopecia, no rash, no oral ulcers, and no heart failure symptoms. On physical examinations, she was febrile with the temperature of 38.2°C and mildly dehydrated, but otherwise comfortable, not tachypneic, and not in distress. Her blood pressure was 128/86 mmHg, and her pulse rate was 110 beats per minute. Her abdomen was soft, not tender, and no palpable organomegaly. Examination of the cardiorespiratory and neurological system was also revealed no abnormal finding. Initial blood investigation was done (as shown in Table 1). Her white blood cells and C‐reactive protein (CRP) were elevated, and there was mild hypoalbuminemia, acute kidney injury, and minimal proteinuria. She was treated as infective acute gastroenteritis with mild degree of acute kidney injury secondary to dehydration and was started on intravenous (IV) ceftriaxone 2 gm once daily. She was also given four pints of IV 0.9% saline over 24 h. In view of the presence of proteinuria, early reactivation of lupus nephritis was suspected. She was given IV hydrocortisone 200 mg three times a day (total dose of 600 mg per day). The oral prednisolone was withheld, while her oral azathioprine and oral hydroxychloroquine were continued. Shortly after the first dose of IV hydrocortisone, she developed severe sinus bradycardia with the heart rate of 30–40 beats per minute. Her blood pressure was 140/70 mmHg. Electrocardiogram (ECG) which was done immediately showed sinus bradycardia with the heart rate of 30 beats per minute, partial right bundle branch blocked, and no evidence of coronary ischemia (as shown in Figure 1). She was put on continuous cardiac monitoring and on 2L oxygen supply via nasal cannula. The IV hydrocortisone was withheld and was switched to IV methylprednisolone 50 mg once daily. Serial cardiac enzyme was taken, and it was not raised. Repeated ECG showed no dynamic ischemic changes with persistent sinus bradycardia. IV atropine was not given yet as she was asymptomatic and her blood pressure was stable. Echocardiography was done and showed normal left ventricular ejection fraction without any regional wall hypokinesia and dilated chambers. Her heart rate slowly improved after the withdrawal of hydrocortisone and become normal after 2 days. There was no similar complication seen with IV methylprednisolone. She was admitted for a total of 3 days. Upon discharge, her symptoms resolved, her renal function, white blood cells, and CRP level normalized, and she was prescribed with oral cefuroxime 250 mg twice daily and oral prednisolone 30 mg once daily, which were to be tapered weekly, along with oral azathioprine 50 mg once daily and oral hydroxychloroquine 200 mg twice daily.

**TABLE 1:** Initial laboratory investigation done upon arrival

Investigations	Result	Normal range
Hemoglobin	11.8 g/dl	12–18 g/dl
Platelet	187 × 10^9^/L	150–400 × 10^9^/L
White blood cell	26.8 × 10^9^/L	4.0–11.0 × 10^9^/L
Albumin	30 g/L	35–50 g/L
Alkaline phosphatase	49 U/L	50–150 U/L
Alanine transaminase	15 U/L	5–35 U/L
Random blood sugar	5.3 mmol/L	<11.1 mmol/L
Creatinine	154.8 μmol/L	60–120 μmol/L
Sodium	135 mmol/L	135–150 mmol/L
Potassium	3.6 mmol/L	3.5–5.0 mmol/L
Urea	10.9 mmol/L	1.7–8.0 mmol/L
Corrected calcium	2.32 mmol/L	2.15–2.55 mmol/L
Phosphate	0.93 mmol/L	0.75–1.50 mmol/L
Magnesium	0.78 mmol/L	0.66–1.07 mmol/L
C‐reactive protein	29.7 mg/dl	0.5 mg/dl
C3 level	102 mg/dl	80–178 mg/dl
C4 level	26.3 mg/dl	12–42 mg/dl
Blood pH	7.38	7.35–7.45
Blood bicarbonate	18.4 mmol/L	21–28 mmol/L
Blood PaCO_2_	28.5 mmHg	35–45 mmHg
Urine FEME	2+	Negative
Urine protein creatinine index	0.06 gm/mmol	0.02 gm/mmol

Note

The white blood cells and C‐reactive protein were elevated, and there was mild hypoalbuminemia, acute kidney injury, and minimal proteinuria. Otherwise, her liver function was normal, there was no electrolyte imbalance and no coagulopathy, and the C3 and C4 levels were normal.

## 3 DISCUSSION

Corticosteroid‐induced bradycardia is a rare condition that only has a few reported cases written in the literature. It was first reported in 1986 and had several hypotheses to explain its occurrence since then. However, the exact pathophysiological mechanism for this adverse effect is still unknown and likely to be multifactorial. One plausible proposed mechanisms is that corticosteroids induce a sudden shift in electrolytes and water physiology, leading to the expansion of plasma volume. This, in turn, activates the low‐pressure baroreceptors and resulted in bradycardia.^1^ Another postulated mechanism is that high‐dose corticosteroid altered the sensitivity of SA node to catecholamine.^2^ According to a study by Vasheghani‐Farahani et al., (2001) which involved 52 hospitalized patients with acute flare of multiple sclerosis and was treated with high‐dose corticosteroids therapy, sinus bradycardia was observed in 41.9% of the patients.^3^ There are several predisposing factors to developing corticosteroid‐induced bradycardia, including a rapid rate of intravenous infusion and the presence of underlying cardiac or renal diseases, and electrolyte imbalances will increase the risk of developing this adverse effect.^4^ Therefore, any electrolyte deficits should be corrected prior to the initiation of treatment.^5^ Our patient has an underlying chronic kidney disease stage 3 due to her lupus nephritis, which may have been the predisposing factor for the sinus bradycardia after she was given a high dose of intravenous hydrocortisone.

Most cases of corticosteroid‐induced sinus bradycardia are asymptomatic.^6^ It is a benign and self‐limiting condition that usually recovered spontaneously after stopping the medication or reducing the corticosteroid doses.^7^ However, it is very important to rule out other more common causes of sinus bradycardia before concluding that it is secondary to corticosteroids. Therefore, a thorough investigation must be performed to rule out systemic causes of sinus bradycardia, which include hypothyroidism, electrolyte imbalance, and the use of other medications that can cause bradycardia such as beta‐blockers, calcium channel blockers, and digitalis. In our patient, her cardiac evaluation was normal; there was no electrolyte imbalance, her thyroid function was normal, and she did not take any medication to reduce her heart rate. The sinus bradycardia episode recovered spontaneously to normal sinus rhythm after the intravenous hydrocortisone was stopped and the episode did not recur when it was changed to intravenous methylprednisolone. There was no recurrent episode of sinus bradycardia with intravenous methylprednisolone and oral prednisolone. She is currently well and continued her long‐term follow‐up under combined nephrology and rheumatology clinic for her systemic lupus erythematosus and lupus nephritis.

## 4 CONCLUSIONS

In conclusion, severe sinus bradycardia can occur as an adverse effect of high‐dose intravenous hydrocortisone, which should be considered as a potential cause of unexplained bradycardia when all other more common causes have been excluded. It is reversible upon stopping the medication and does not recur with other types of corticosteroids.

## CONFLICTS OF INTEREST

The authors have no potential conflicts of interest to disclose.

## AUTHOR CONTRIBUTIONS

Alvin Oliver Payus wrote the manuscript, edited the final version, and become correspondence. Chai Ann Chee collected the data and wrote the manuscript. Norlaila Mustafa edited the final version and become supervisor.

## ETHICAL APPROVAL

The authors have obtained the informed written consent for writing and publishing this article from the patient.

## Data Availability

Data sharing not applicable to this article as no datasets were generated or analysed during the current study.
